# Organization-level determinants for low secondary traumatic stress in lay counselors delivering trauma-focused cognitive behavioral therapy in Kenya

**DOI:** 10.1371/journal.pgph.0006360

**Published:** 2026-05-26

**Authors:** Priya Dahiya, Clara Johnson, Rashed AlRasheed, Christine L. Gray, Rosemary Meza, Kathryn Whetten, Daisy Anyango Okoth, Omariba Anne Nyaboke, Shannon Dorsey

**Affiliations:** 1 Department of Psychology, University of Washington, Seattle, Washington, United States of America; 2 Department of Community Medicine and Behavioral Sciences, College of Medicine, Kuwait University, Kuwait City, Kuwait; 3 Center for Health Policy and Inequalities Research, Duke Global Health Institute, Duke University, Durham, North Carolina, United States of America; 4 Kaiser Permanente Washington Health Research Institute, Seattle, Washington, United States of America; 5 Sanford School of Public Policy, Duke University, Durham, North Carolina, United States of America; 6 Trauma Focused Cognitive Behavioral Therapy Train-the-Trainer Training and Mental Health Consultation, Bungoma, Kenya‌‌; University of California San Francisco, UNITED STATES OF AMERICA

## Abstract

Eighty percent of the world’s youth live in low- and middle-income countries (LMICs), yet access to trauma-focused mental health care in these settings remains limited despite a high burden of mental health disorders and trauma exposure among youth. Task-shifting models that train lay counselors to deliver evidence-based treatments can expand access to care, yet delivering trauma-focused treatment may increase counselors’ vulnerability to secondary traumatic stress (STS). Organization-level support may play an important role in sustaining counselor well-being, yet little is known about which organizational factors are protective (i.e., contribute to low STS) in resource-limited settings. Data came from an implementation-effectiveness trial for an adapted trauma-focused cognitive behavioral therapy in western Kenya, to examine organizational factors linked to low STS among two groups of lay counselors: community health volunteers (CHVs; N = 120) and teachers (N = 117). Counselors completed surveys following training and treatment delivery that assessed supervisory relationships, leadership, implementation climate, feasibility, and organizational climate. We applied Coincidence Analysis, a configurational method, to identify organization-level determinants of low STS. Among CHVs, a solution with three pathways was identified for low STS: high supervision relationship; high implementation climate with high implementation leadership; and high feasibility with high transactional leadership. Among teachers, a different solution with three pathways emerged: high implementation climate; high supervisory relationship with a positive organizational climate via perceived work environment; and low transactional leadership with high transformational leadership. No single organizational factor was necessary for low STS. Instead, multiple configurations were sufficient, and these differed across provider roles and their organizational contexts. These findings suggest that organizational strategies to prevent or mitigate STS should be tailored to provider roles and settings. As task-shifting models expand in LMICs, centering lay counselors’ well-being within organizational management and treatment development will be critical to sustaining the workforce and ensuring quality trauma-focused care.

## Introduction

Mental health problems are the leading cause of disability among youth [1]. Although 80% of all youth live in low- and middle- income countries (LMICs) [2], access to evidence-based mental health care in LMICs remains low due to limited mental health funding [3] and few mental health professionals [4]. Effective, trauma-focused mental health services are especially needed in LMICs as rates of trauma exposure and mental health impact in youth are high [5]. One solution to scaling up mental health services – such as trauma-focused services – in LMICs is the use of task-shifting, or task-sharing [6,7], which often involves training lay counselors with minimal formal mental health experience in the delivery of mental health services under supervision. Due to limited specialized health care settings in LMICs, lay counselors often come from *non-traditional care settings* (e.g., schools, non-governmental organizations, faith or religion-based organizations, community-based organizations) and assume responsibility of providing mental health care for clients [8,9]. Several studies have demonstrated that lay counselors can expand access to mental health services, including trauma-focused services, in LMICs [10,11] and that services delivered by a lay counselor can be effective when counselors have adequate training and supervision and engage in collaborative care [12].

While task-shifting can increase access to trauma-focused services in LMICs, leveraging lay counselors to build capacity partially addresses the treatment gap in LMICs. Organizational characteristics such as availability of time and resources (e.g., funding and location availability), formal training, and regular supervision are important contributors to successful implementation of trauma-focused interventions in LMICs [13,14]. Some studies have found that organizational culture and climate as well as leadership support facilitated successful implementation of trauma-focused interventions [15,16], though these studies were mainly based in western high-income countries and involved trained mental health clinicians, not lay counselors. Given the limited research on organizational factors that support task-shifting in LMICs, it is important to consider context-specific organizational factors that support lay counselors’ trauma-focused service delivery in non-traditional health care settings.

While there is a need to improve access to trauma-focused services for youth, delivering trauma treatment poses unique challenges to providers, including the risk of developing secondary traumatic stress (STS). STS develops in response to indirect exposure to traumatic events, and symptoms of STS parallel those of post-traumatic stress disorder (e.g., psychological distress, hyperarousal, insomnia, etc.) [17–19]. This exposure often occurs in the context of supporting survivors of trauma within a professional work setting. Thus, those who work directly with individuals exposed to traumatic stressors are at a relatively high risk for developing STS [20]. STS is a common experience for many mental health professionals, with upwards of 40% of providers reporting STS in different settings [21,22]. Lay counselors face heightened risk of developing STS due to compounding stressors like high caseloads paired with limited training, which can compromise their own well-being and the quality of care they provide.

Prior work largely highlights individual-level factors that predict STS such as personal history of trauma or high intensity and frequency of secondary exposure [23–26]. Relatedly, prior work also identifies individual-level factors that either protect against the development of STS or reduce existing symptoms on a personal level. These include strategies such as interpersonal social support from friends, family, or co-workers, mindful self-care, self-compassion, cognitive coping, and other trauma-focused treatment approaches [27–30]. STS has historically been described as an individual issue for providers to manage in response to prolonged exposure to traumatic experiences. While individual strategies like self-care or social support are important, they place the responsibility for managing symptoms entirely on the provider, adding additional burden and effort to already overwhelmed providers and potentially increasing their vulnerability [31]. Moreover, STS often does not occur in a vacuum as it is shaped by the systems and environments counselors are working in.

Thus, recent work has also considered risk and protective factors on a more macro-level (i.e., organizational factors). Workplace organizational factors such as high workload and lack of practical trauma-specific training can contribute to STS development [32]. A 2020 study in the Western Cape province of South Africa reported that 50% of lay counselors were at significant risk of adverse psychological outcomes from elevated levels of STS, potentially due to high caseloads and limited training, and echoed a need for STS education and peer or supervision support [30]. Another study conducted in Bukavu in the Democratic Republic of Congo (DRC) found, through qualitative interviews, that a lack of formal knowledge about STS, limited supportive tools like supervision, and the nontraditional work environment in post-war DRC (i.e., poverty, limited access to work training, and political instability), among other factors, played major roles in STS severity [33]. Research is limited on organization-level factors associated with low STS among lay counselors in particular. However, among other mental health professionals, factors like supervision, supportive leadership, respectful and collaborative work environments, and support at the peer-, managerial-, and institutional-level are tied to low workforce STS [34–36]. Other workplace-specific factors that decrease the likelihood of STS include environments with effective communication and collaboration, appropriate staffing, staff recognition and appreciation, caring leadership, professional development, and lower mistrust and intimidating behavior [37,38]. Focused attention on STS in organization-wide efforts has been shown to improve provider outcomes [39]. One area of research that, to our knowledge, is yet to be explored is the relationship between implementation climate– organizational members’ shared perception that a particular intervention is expected, supported, and rewarded [40] – and STS. A positive implementation climate of a given intervention is theorized to promote skillful, enthusiastic, and committed use of such given intervention [41], which in combination with supportive leadership and/or positive work environment, may facilitate successful intervention implementation and be protective against STS. Johnson & colleagues found that implementation climate and implementation leadership were important determinants of TF-CBT feasibility delivered by teachers (i.e., lay counselors) in schools in Kenya [42]. Providers’ perception of how easy it is to deliver a trauma-focused treatment in their work setting (i.e., feasibility of treatment implementation) is an important factor to consider because experiencing difficulty in delivering a trauma-focused treatment may increase risk of STS, especially in a context of competing demands and limited supports.

By focusing on the organizational context, the question shifts from *“What should counselors do?”* to *“What can organizations do to protect their workforce?”*, which is especially important in resource-limited settings like LMICs, where lay counselors often face high demands with limited institutional support. This area of work has mainly been conducted in western settings, and little is known about how organizational factors operate in settings with lay counselors and non-traditional care structures. Understanding STS prevention and mitigation in these contexts is essential for developing strategies that sustain lay counselors in delivering trauma-focused interventions. Considering the critical role lay counselors play in access to care, their well-being is crucial to ensuring children continue to receive high-quality mental health care. Focusing on organization-level factors rather than individual resilience alone allows for organization-level, long-term solutions to help counselors remain engaged in care delivery.

The present study builds upon the existing literature on organizational factors related to STS in the mental health workforce. Prior studies with mental health professionals suggest that peer and leadership support, collaborative work environments, high-quality supervision, and other related factors can shape the risk of STS. The current study adds to existing literature by exploring factors that are determinants of low STS among lay counselors in western Kenya. Teachers and Community Health Volunteers (CHVs) delivered a culturally adapted version of trauma-focused cognitive behavioral therapy (TF-CBT) to youth in school settings. In addition to supervision and leadership support, we anticipated that implementation leadership and climate will also play a role considering the context of implementing treatment in a non-specialty mental health setting. Feasibility—counselors’ perception of how easy it is to deliver TF-CBT—may also play a role in influencing STS especially in these settings with high demands and limited resources. Using a configurational comparative method called Coincidence Analysis, we conduct exploratory analyses to examine pathways to low STS using the following organizational factors: implementation climate, implementation leadership, supervisory relationship, feasibility, organizational climate via perceived work environment or personal feelings at work, transactional leadership, and transformational leadership.

## Methods

### Parent study: Trial description

The present study is a secondary analysis of data from Building and Sustaining Interventions for Children (BASIC): Task Sharing Mental Health Care in Low-Resource Settings [43], which is a Hybrid Type II Implementation-Effectiveness Trial of Pamoja Tunaweza (PT), “Together We Can” in Kiswahili, a culturally-adapted version of Trauma-Focused Cognitive Behavioral Therapy (TF-CBT) [44] for youth. PT was originally developed through a long-standing partnership among Ace Africa, a Kenyan non-governmental organization, Tanzania Women’s Research Foundation, and two U.S. universities. The intervention has been found to be effective in reducing post-traumatic stress and prolonged grief symptoms among children in Kenya [45,46]. The BASIC study was designed to identify implementation policies and practices that lead to the effective implementation and sustainment of PT across two governmental sectors identified by Ace Africa as critical for mental health care delivery for youth in Kenya: Education (via teachers) and Health (via CHVs). CHVs primarily extend health services from health facilities to communities under the supervision of community health extension workers.

The trial was conducted across 40 sites, each with a primary school and a health extension that formed a village cluster to include both a team of teachers and a team of CHVs delivering the group-based PT intervention. Children ages 11–14 who had experienced parental death and demonstrated posttraumatic stress symptoms and/or prolonged grief were identified for the study. Participants were randomized to receive PT from either teacher-counselors or CHV-counselors. Each counselor team conducted two sequential 8-week PT groups, one for boys and one for girls (in different orders), ultimately providing PT to 849 children across all study sites.

The BASIC trial design was a stepped-wedge cluster-randomized trial, and implementation occurred in order across seven sequences to which the 40 sites were randomly allocated. Sites in each of sequences 2–7 functioned as comparison sites for the prior sequence (e.g., Sequence 2 as a comparison for Sequence 1), continuing with existing school and community practices until their assigned implementation sequence began. When a sequence entered its implementation phase, the counselors from all sites in that sequence were trained and began delivering PT, supported by ongoing supervision and fidelity monitoring. Experienced teachers and CHVs from the first sequence co-designed and provided low intensity implementation support (i.e., coaching) to counselors in the remaining sequences. Coaching involved meeting across four implementation phases to design a workplan through which newly trained counselors could identify solutions for perceived determinants (e.g., leadership support, obtaining needed resources [chalk, paper] in advance).

PT training was led by Kenyan lay counselor PT experts, who first deliver PT with supervision and subsequently participated in a Train-the-Trainer model to develop training expertise for BASIC [47]. They provided 5–6 days of structured training that included didactic instruction, experiential activities, modeling, small group role-plays, and structured feedback from trainers and peers. After completing training, counselors received weekly supervision from these PT experts during the initial implementation phase, which transitioned to monthly supervision in the sustainment phase. Fidelity was assessed in two ways. Counselors completed structured session reports documenting attendance, session content, and participant engagement. PT expert supervisors conducted direct observations twice per month during the first year and monthly observations in the sustainment phase to provide implementation support and feedback. Counselors completed quantitative surveys after PT training and after PT implementation with trained interviewers in person.

### Ethics statement

The Duke University Institutional Review Board (IRB) and the Kenya Medical Research Institute Scientific and Ethics Review Unit (SERU) oversaw and granted approval for BASIC (R01MH112633). The recruitment period for the participants in the present study was from 2/2/2018–1/17/2022. Informed consent was provided verbally and in writing by participants, and interviewers administering the consent also witnessed and co-signed consent documents.

### Parent Study: Intervention

Pamoja Tunaweza (PT) was a group-based, 8-session intervention. The intervention includes core TF-CBT components (e.g., psychoeducation, parenting, relaxation, affective modulation, cognitive coping, in vivo exposure planning, and grief-specific skills) [44]. Imaginal exposure and some child-specific cognitive restructuring were conducted mid-group using 1–2 additional, individual sessions. Cultural adaptations in the prior pilot [46] and randomized controlled trial [43] were made in close collaboration with Kenyan providers and included reframing the intervention as a “class” to reduce stigma, modifying language and examples to reflect local experiences, and structuring sessions for group delivery. Each PT group included weekly child sessions and concurrent guardian sessions, and joint child-guardian activities for the final four PT group sessions. All PT groups, regardless of counselor type, were delivered in schools as school leaders offered the school building, which served as a neutral community space for Health sector delivery in communities, in addition to Education sector delivery. The only deviation from this protocol occurred during the COVID-19 pandemic, when groups were temporarily paused.

### Present Study: Secondary sata analysis

The present study examines organizational factors tied to low STS among teacher-counselors and CHV-counselors delivering PT. Data come from counselor self-report quantitative surveys at two BASIC data collection points, following PT training and after PT implementation. Trained interviewers conducted verbal interviews using Qualtrics. All study measures were available in Kiswahili and English; interviewers were also fluent in both languages. Counselors could choose which language they preferred for their interview. Teachers often chose English; CHVs often chose Kiswahili.

Participants: The secondary data analysis utilizes data from 237 counselors: teacher-counselors (N = 117) or CHV-counselors (N = 120). Teachers were embedded within the education sector while CHVs were embedded in the health sector. However, both Teachers and CHVs delivered PT within government schools, where they held different roles and identities. Teachers held full-time, paid positions within their school and had competing demands on their time, like lesson planning, teaching, and other school roles (e.g., school clubs). CHVs were engaged in part-time voluntary work under the supervision of their Community Health Extension Workers focused on health promotion in communities, extending the reach of their health systems (e.g., sanitation programs, bed-net distribution). CHVs were not embedded in the school organizational context as they were visitors who traveled to schools to provide treatment. Given differences in teacher and CHV organizations and work, challenges and support captured in organization-level factors may have differed among these counselors. Thus, CNA analyses were conducted separately to identify solutions that uniquely serve both sets of counselors.

Measures: When possible, the study team used validated measures from prior work in Kenya or measures already used in Kenya, if not yet validated. For constructs where measures were not already used or validated in Kenya, the study team followed best practices in translation and cultural adaptation [48], including orienting Ace Africa research staff to each construct and then reviewing and discussing all questions on each measure in plain language [49] to ensure that each question itself made sense in the local context. Then measures were translated to Kiswahili and back translated (different translator). Lastly, meaning was checked/refined collaboratively based on the back translation by Ace Africa staff, with further revisions, translation, and back translation as needed. [50,51]

The primary outcome variable, STS, was measured cross-sectionally post intervention and included items measuring distress tied to client trauma (e.g., I am not as productive at work because I am losing sleep over traumatic experiences of a child/guardian I counseled in Pamoja Tunaweza/TF-CBT”). A detailed overview of the outcome variable and organizational factors used in these analyses, including definitions, measurement scales, and psychometric properties, is provided in Table 1.

**Table 1:** Outcome and Determinant Measures.

Construct	Measure	Definition	Scale	Measure Properties
Secondary Traumatic Stress (STS)	Professional Quality of Life Compassion Fatigue subscale	Counselors’ emotional exhaustion tied to client trauma.	3 items rated on a 5-point Likert Scale from 1 (*Never*) to 5 (*Always*); higher scores indicate higher STS. Example item: “*I am not as productive at work because I am losing sleep over traumatic experiences of a child/guardian I counseled in PT.*”	Prior research demonstrated that the STS subscale had a good internal consistency (ɑ = 0.81) [52]. In our sample, this measure demonstrated acceptable internal consistency given its brevity (α = .67) and a moderately strong average inter-item correlation (IIC = .41).
Implementation Climate	Implementation Climate Scale (ICS)	Counselors’ perception of the degree to which PT was expected, supported, and rewarded in their organization.	10 items rated on a 5-point Likert Scale from 1 (Strong Disagree) to 5 (Strongly Agree); higher scores indicate a more positive implementation climate. Example item: *“I am expected to use PT with children at my school.”*	Prior research demonstrated ICS’s excellent overall internal consistency (ɑ = 0.91), and reliable organizational-level means (ICC = 0.25). In our sample, this measure demonstrated acceptable internal consistency (α = .70) and an average inter-item correlation within the recommended range (IIC = .37).
Implementation Leadership	Implementation Leadership Scale (ILS)	Counselors’ perceptions of leadership support for PT implementation.	12 items rated on a 5-point Likert Scale from 0 (*Not at All*) to 4 (*Very Great Extent*); higher scores indicate Higher scores indicate stronger implementation leadership. Example item: *“[Leader] has removed obstacles to the implementation of PT.”*	Prior research found excellent internal consistency (ranging from α = 0.95–0.98) and acceptable reliability of organizational-level means (ICC = 0.29) for the original measure [53]. In our sample, this measure demonstrated excellent internal consistency (α = .93) and a high average inter-item correlation (IIC = .53).
Organizational Climate via Personal Feelings at Work	Organizational Climate Personal Feelings at Work subscale	Counselors’ perceptions of how they feel at work.	12 items rated on a 5-point Likert Scale from 0 (*Never*) to 4 (*Always*); higher scores indicate more positive feelings at work. Example item: *“Does the school for which you work promote your professional growth?”*	Prior research demonstrated that the Personal Feelings at Work subscale had good internal consistency (ɑ > 0.80) [54]. In our sample, this measure demonstrated acceptable internal consistency (ɑ = 0.72) and a low average inter-item correlation (IIC = .15).
Organizational Climate via Perceived Work Environment	Organizational Climate Perceived Work Environment subscale	Counselors’ perceptions of their work environment.	4 items rated on a 5-point Likert Scale from 0 (*Never*) to 4 (*Always*); higher scores indicate more positive perceptions of the work environment. Example item: *“Do you feel that there is a high level of cohesion within your school?”*	Prior research demonstrated that the Perceived Work Environment subscale had good internal consistency (ɑ > 0.80) [54]. In our sample, this measure demonstrated acceptable internal consistency acceptable internal consistency (α = .71) and a moderate average inter-item correlation (IIC = .38).
Supervisory Relationship	Supervisory Relationship Questionnaire	Counselors’ perception of whether supervisors provided a collaborative and safe environment to discuss clinical cases, reflective and educational support during supervision, and the prescribed elements of supervision.	14 items rated on a 7-point Likert Scale from 1 (*Strongly Disagree*) to 7 (*Strongly Agree*); Higher measure scores indicate a stronger perceived supervisory relationship by counselors. Example items: *“My supervisor was approachable”, “My supervisor encouraged me to self-evaluate my delivery of PT”, “Supervision meetings were focused.”*	Prior research demonstrated each subscale to have good internal consistency (α = 0.88-0.97). The overall measure had strong test–retest reliability (r = 0.94) [55]. In our sample, this measure showed good internal consistency (α = .86) and average inter-item correlation within the recommended range (IIC = .33).
Feasibility	Johns Hopkins University (JHU) Feasibility scale	Counselors’ perception of the feasibility of delivering PT	12 items rated on a 5-point Likert Scale from 1 (Strong Disagree) to 5 (Strongly Agree); higher scores indicate stronger perceived feasibility. Example items: *“I am provided with the necessary transportation to regularly provide PT”, “I have access to the emotional support I may need to handle any stress related to delivering PT (e.g., hearing children’s stories about their parent(s’) death).”*	Prior research demonstrated that the JHU feasibility scale had good internal consistency (ɑ = 0.85) [56]. In our sample, this measure demonstrated good internal consistency (ɑ = 0.82) and inter-item correlations within the recommended range (IIC = 0.30).
Transactional Leadership	The Multifactor Leadership Scale Transactional Leadership subscale	Counselors’ perception of the extent to which their organizations demonstrate transactional leadership (consequence and rewards-motivated).	12 items rated on a 5-point Likert Scale from 1 (*Never*) to 5 (*Always*); higher scores indicate higher transactional leadership. Example item: *“How often does [leader] express satisfaction when you meet expectations?”*	Prior research demonstrated that the Multifactor Leadership transactional leadership subscale has acceptable to good internal consistency (ɑ = 0.69-0.90) across studies [57,58]. In our sample, this measure demonstrated marginal internal consistency (α = .68) and a low-to-moderate average inter-item correlation (IIC = .16).
Transformational Leadership	The Multifactor Leadership Scale Transformational Leadership subscale	Counselors’ perception of the extent to which their organizations demonstrate transformational leadership (supportive, encouraging, inspirational).	20 items rated on a 5-point Likert Scale from 1 (*Never*) to 5 (*Always*); higher scores indicate higher transformational leadership. Example item: *“How often does [leader] specify the importance of having a strong sense of purpose?”*	Prior research demonstrated that the Multifactor Leadership transformational leadership subscale has good to excellent internal consistency (ɑ = 0.85-0.94) across studies [57,58]. In our sample, this measure demonstrated good internal consistency (α = .88) and a moderate average inter-item correlation (IIC = .27).

### Analytic plan

A major challenge within implementation science is utilizing an analytic method that aligns with applied settings and the nuance of applied data. To address this challenge, the present study applies Coincidence Analysis (CNA) to identify which organizational factors make a difference in whether lay counselors delivering PT in Kenya experience low levels of STS. CNA is a configurational comparative method based in Boolean algebra that identifies necessary and sufficient conditions (i.e., a factor-value) and/or configurations (i.e., combinations of conditions) [59,60]. Unlike traditional variable-centered methods like regression models that assess individual variables’ net effects, CNA examines how different configurations of conditions can produce an outcome and explores relationships between different factors by analyzing how frequently they occur together leading to the presence or absence of a certain outcome. In other words, CNA allows us to identify conditions of organizational factors, and configurations of those conditions that distinguish STS severity. Given the limited prior work specifying which organizational factors are most relevant in this particular context, no a priori hypotheses are specified for these analyses.

CNA is a practical analytic approach in that it allows for the possibility of equifinality—that different configurations may result in the same outcome and is redundancy free in identifying the minimal set of necessary and sufficient conditions for an outcome to occur. These qualities are especially useful in resource-limited settings because organizations can prioritize the most impactful and least intensive factors that determine low STS.

The analysis followed the steps for CNA described in Baumgartner and Ambühl’s (2020) guide for the “cna” function in R [61] and data deduction approaches in the CNA literature [42,62]. We selected organizational factors hypothesized to be associated with STS for the analyses. We first identified organizational factors based in theory and literature on predictors of STS. Prior work suggests that workplace culture, leadership, structural and social support, and positive supervisory relationships, among other factors, play a role in the frequency and intensity of STS that counselors experience. As described in the introduction, we started with 8 factors that were collected as a part of the parent study: implementation climate, implementation leadership, supervisory relationship, feasibility, organizational climate via perceived work environment or personal feelings at work, transactional leadership, and transformational leadership.

In CNA, continuous factors are categorized to set membership scores (i.e., dichotomous variables in crisp-set CNA) through a process called calibration to identify how factor values (i.e., conditions) work in combination with other conditions to produce a specific outcome. In conducting a crisp-set CNA, all 8 continuous factors were calibrated into binary conditions based on both theoretical assumptions and the distribution of the data, following CNA best practices. In regression-based analyses, interpretation of factor scores occurs following the analysis, which differs from the proactive CNA approach in which interpretation occurs prior to analysis. Factors were calibrated to maximize variance between 30–70% whenever possible. The outcome variable (STS) was dichotomized from a 1 (never)-5 (always) scale to two conditions: low STS (scores ≤2 – never or rarely experienced) and moderate/high STS (scores > 2—sometimes, often, always experienced). This dichotomization process was repeated for each factor, based on the distribution of scores for each factor and practical differences. The same calibration was used for teacher and CHV analyses to ensure comparability in data interpretation (see Table 2 for calibration thresholds and final binary distributions).

**Table 2:** CNA Calibrations of Determinants and Outcome Variables.

Variable	Range	Calibrations	Calibration Split (Teachers)	Calibration Split (CHVs)
Secondary Traumatic Stress (Outcome)	1–5	Low: < 2Moderate/High: 2–5	85 / 117 (72.6%)	102 / 120 (85%)
Implementation Climate	1–5	Low/Moderate: < 4.25 High: 4.25 - 5	53 / 117 (45.3%)	77 / 120 (64.2%)
Feasibility	1–5	Low/Moderate: < 4.25 High: 4.25 - 5	51 / 117 (43.6%)	69 / 120 (57.5%)
Implementation Leadership	1–4	Low/Moderate: < 3 High: 3–4	37 / 117 (31.6%)	65 / 120 (54.2%)
Supervisory Relationship	1–7	Low/Moderate: < 6 High: 6–7	57 / 117 (48.7%)	62 / 120 (51.7%)
*Organizational Climate*				
Personal Feelings at Work	1–5	Low/Moderate: < 3 High: 3–5	49 / 117 (41.9%)	50 / 120 (41.7%)
Perceived Work Environment		Low/Moderate: < 3.5 High: 3.5 - 5	52 / 117 (44.4%)	61 / 120 (50.8%)
*Multifactor Leadership*				
Transactional	1–5	Low: < 2.5 Moderate/High: 2.5 - 5	60 / 117 (51.3%)	58 / 120 (48.3%)
Transformational		Low/Moderate: < 3 High: 3–5	66 / 117 (56.4%)	80 / 120 (66.7%)

Once calibrated, we conducted a “minimally sufficient conditions" (msc) routine in the R package “cna” [63] to the full dataset, an established routine used to reduce data including in the final analysis [64,65]. The msc routine includes running multiple CNA with various fit thresholds (consistency and coverage described below) and identifies 1-, 2-, and 3-condition configurations that appear throughout the CNA output. These conditions are considered to be connected to the outcome and important to include in the final CNA. After these were identified, CNA was performed to identify pathways conditions and configurations that are sufficient and/or necessary for low STS. Consistency and coverage are used to evaluate sufficiency and necessity in CNA. *Consistency* refers to how frequently a condition or configuration is present when the outcome of interest occurs, among all cases when the condition/configuration is present. *Coverage* indicates how frequently a condition or configuration occurs, among all cases when the outcome occurs. Due to high prevalence in the outcome (72.6% teachers and 85% of CHVs experienced low STS), utilizing standard consistency and coverage measures would likely inflate results. Instead, sufficiency was assessed using prevalence-adjusted consistency (PA-consistency) and necessity through prevalence-adjusted contrapositive coverage (PA-coverage) which address limitations of a high outcome prevalence [66]. Following best-practices, we initially set PA-consistency and PAC-coverage thresholds to 0.75. For CHV delivery, consistency and coverage remained at 0.75; however, due to data imbalance among teachers, we reduced the PA-consistency and PAC-coverage thresholds to 0.70. While lower thresholds can indicate risk of underfitting, this range is consistent with prior CNA studies and falls within the recommended threshold range of 0.70-1 [42,66].

Other fit measures were also used. Specifically, the fit-robustness score (frscore) from the R package cna was applied to mitigate overfitting and to further assess model ambiguity when multiple solutions exhibited similar consistency and coverage scores. We ran models across a range of consistency and coverage thresholds from .70 to 1.0 and prioritized solutions that were practical and applicable to the research context that had a high frscore [67]. In addition, we prioritized solution pathways that had lower complexity (i.e., fewer configurations present), and higher faithfulness and exhaustiveness, two measures of correspondence between expected configurations that are compatible with the solution and actual configurations produced in the data that informed model results [66].

We conducted two exploratory CNA analyses to identify difference making conditions for low levels of STS among both counselor types.

CNA Analysis 1: Organizational conditions that are necessary and/or sufficient for low STS in teacher-counselors.

CNA Analysis 2: Organizational conditions that are necessary and/or sufficient for low STS in CHV-counselors.

## Results

### Lay counselor descriptives

Among teachers, 65.0% were female (n = 76), with a mean age of 41.9 years (SD = 7.8) and an average of 15.6 years (SD = 8.6) of teaching experience. The most commonly reported highest level of education was a diploma certificate, held by 52.9% of teachers. CHVs were also predominantly female (69.2%, n = 83), with a mean age of 43.7 years (SD = 10.3) and an average of 7.5 years (SD = 4.7) of experience as CHVs. The most common highest level of education among CHVs was secondary school (equivalent to high school in the US), reported by 69.1% of participants.

### Difference makers for low STS: CNA results

Descriptive statistics for outcome and determinants are described in Table 3.

**Table 3:** Descriptive Statistics for Outcome and Determinants.

Variable M (SD)	Teachers (*N* = 117)	CHVs (*N* = 120)
Secondary Traumatic Stress	1.66 (0.56)	1.26 (0.46)
Supervisory Relationship	5.94 (0.37)	5.98 (0.38)
Implementation Climate	4.27 (0.47)	4.49 (0.42)
Implementation Leadership	2.59 (0.74)	3.04 (0.67)
Feasibility	4.21 (0.42)	4.32 (0.39)
Organizational Climate via Personal Feelings at Work	2.86 (0.47)	2.87 (0.43)
Organizational Climate via Perceived Work Environment	2.92 (0.84)	3.25 (0.66)
Transactional Leadership	2.96 (0.58)	2.42 (0.62)
Transformational Leadership	2.44 (0.47)	3.15 (0.60)

Note: Score ranges for measures are found in Table X.

M = Mean; SD = Standard Deviation.

**Fig 1:**
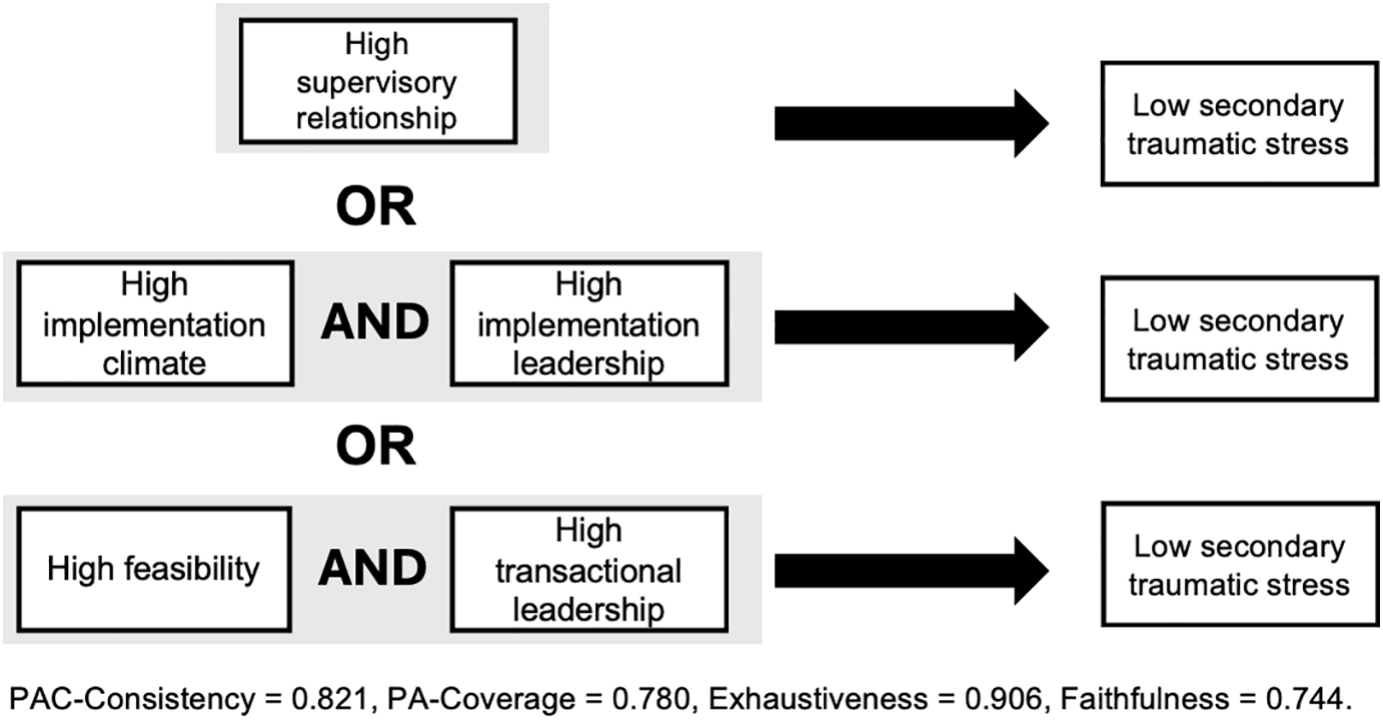
CHV-Counselor CNA Solution Pathways for Low Secondary Traumatic Stress.

Among CHV-counselors, analyses reveal a solution with three disjunctive pathways (i.e., three distinct conditions or configurations) that are sufficient for low levels of STS (PAC-consistency = 0.82, PA-coverage = 0.78, exhaustiveness = 0.91, faithfulness = 0.74). The model is presented in Fig 1.

Pathway 1: If the supervisory relationship scale was high among CHVs, then STS was low.

Pathway 2: If CHVs reported high implementation climate AND high implementation leadership, then STS was low.

Pathway 3: If CHVs reported high feasibility of delivering PT AND high levels of transactional leadership within their organization, then STS was low.

**Fig 2:**
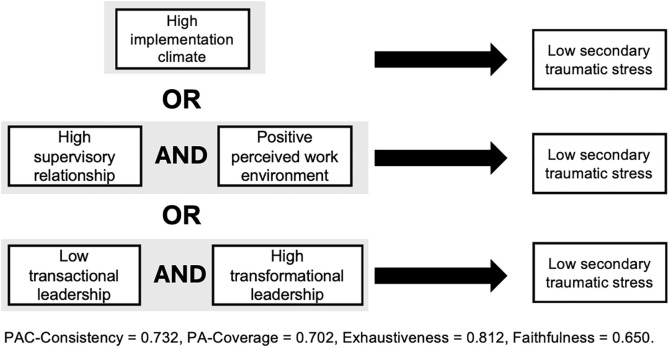
Teacher-Counselor CNA Solution Pathways for Low Secondary Traumatic Stress.

Among teacher-counselors, analyses also reveal a solution with three disjunctive pathways sufficient for low levels of STS (PAC-consistency = 0.73, PA-coverage = 0.70, exhaustiveness = 0.81, faithfulness = 0.65). The model is presented in Fig 2.

Pathway 1: If teachers reported high implementation climate, then STS was low.

Pathway 2: If the supervisory relationship was high AND the organizational climate via perceived work environment was high among teachers, then STS was low.

Pathway 3: If teachers reported low transactional leadership AND high transformational leadership within their organization, then STS was low.

## Discussion

This study examined organization-level factors that make a difference in whether two groups of lay counselors in Kenya—teachers and community health volunteers (CHVs)— experienced low levels of STS when implementing a trauma-focused intervention (PT). It is important to note that prior research suggests that STS is common among providers, with rates up to 39% [21] and 50% among lay counselors in one study [30]. In the present study, however, teacher and CHV counselors delivering the PT intervention had relatively low rates of STS, with only about 20% of the sample experiencing moderate-high STS. While the reasons for lower rates are unclear, counselors in this study were provided with rigorous PT training and ongoing supervision and reported high ratings on organizational factors, which may have contributed to a supportive environment that buffered counselors from STS. This interpretation aligns with prior research emphasizing the importance of supervision in task-shifting models [68] and the role of organizational support in effective task-shifted care [69].

Using CNA, we identified pathways that were sufficient to account for low STS. Findings indicated that organization-level factors can make a difference in counselors’ STS experiences, but these pathways differ based on counselors’ roles and organizational contexts.

### CHVs

For CHVs, our analyses suggest that supervision, PT-specific organizational support and leadership, and structure in logistics and leadership were important to the occurrence of low STS. The first pathway suggests that a high-quality supervisory relationship alone is sufficient to account for low STS. A strong supervisory relationship is characterized by counselors perceiving they were part of a collaborative and safe supervision environment, they received reflective and educational support during supervision, and supervision was well structured. This aligns with prior work emphasizing the role of the supervisory relationship in low secondary trauma among clinicians [35] and lay providers [70,71], particularly the function of supervision that targets counselors’ own emotional experiences and their clinical skills [72,73]. Previous work has shown that counselors’ perception of their clinical skills or ability to deliver treatment is strongly correlated with low STS [74], and supervisory relationships that normalize counselor emotions decreases vicarious trauma risk [75,76]. A 2024 study in Norwegian Child Advocacy Centers found that supervision predicted low STS among mental health providers, over and above all other organizational factors [77]. It may be that the supervisory relationship plays a dual protective role for CHVs—building clinical confidence and processing the emotional impact of trauma-focused treatment. This relationship alone may be particularly valuable to CHVs, who have limited formal training and intervention support beyond the supervisory relationship.

The second pathway for CHVs highlighted the combination of high implementation climate and high implementation leadership was sufficient for low STS. Implementation climate refers to whether PT was expected, supported, and rewarded in their organization and implementation leadership measures strategic leadership support for PT [78]. Together, these factors suggest that CHVs experienced lower STS when they perceived strong organizational buy-in for the intervention from their Community Health Extension Workers (i.e., leaders) and felt those leaders were engaged in supporting implementation efforts (e.g., encouragement, planning, problem-solving). This alignment between expectations, recognition, and leadership action may have created a sense of structure and shared purpose around implementing PT, buffering CHVs from challenges of adding PT to their volunteer work and the burden of trauma-focused treatment by creating an environment that supports their PT delivery. Prior work has suggested that when providers receive consistent implementation support from leaders and organizations, they have a more motivated or positive attitude [79,80] towards treatment as high implementation leadership and climate signals to counselors that treatment is of high priority to their organization.[81] Recent work has explored strategies to improve implementation climate and leadership, including a pre-post outcome evaluation study for a program called Training in Implementation Practice Leadership (TRIPLE) [82] and randomized control trials on the use of Leadership and Organizational Change for Implementation (LOCI) [83,84]. In BASIC, the coaching received by sites in sequences two-seven may have helped organizations focus on how leaders could support their counselors in delivering PT.

The third pathway for CHVs reveals that a combination of high feasibility and high transactional leadership was sufficient for low STS. Feasibility refers to not only counselors’ confidence in their ability to deliver PT but also their access to time, transportation, supervision, emotional support, and physical resources like private space or materials. Transactional leadership refers to a leadership style that prioritizes structure, clear expectations, and reward-based motivation. A pathway with both of these factors suggests that STS was low when CHVs felt both logistically supported to carry out their responsibilities and guided by structured, task-oriented leadership, which aligns with prior work across settings that emotional overwhelm is lower when workers operate in a structured environment [85–87]. For CHVs, this combination of feasibility and transactional leadership may reflect an environment where expectations are clearly communicated and operational needs are met (e.g., sometimes reporting in on a different day given their PT schedule), allowing counselors to focus on their clinical role without the added strain of navigating unclear expectations from leadership. This is especially relevant for CHVs considering that they are not full-time workers or employees of schools where they delivered PT, potentially leading to more logistical challenges (e.g., transportation) than teachers.

Compared to teachers, CHVs are less embedded day-to-day in an organizational structure and more integrated into their communities as “the last mile” of healthcare [88]. Additionally, they deliver care in communities, and in this study, the community setting was the school. Moreover, given that there were no PT activities in the health facilities, CHVs’ site-leaders were minimally involved in the daily implementation of PT compared to teachers’ site leaders.

### Teachers

Teachers, who delivered treatment within their organization/place of employment had organization-level pathways to low STS that overlapped with but differed from CHV-counselors. The first pathway for teachers indicated that positive implementation climate alone was sufficient for low STS. This suggests that when teachers perceived strong support from their organization to deliver PT—such as clear expectations, rewards, support, and resources—STS was lower. Given that teacher-counselors often balanced multiple roles, including teaching and other school duties, there may be a risk of role ambiguity, which is tied to work disengagement [89]. A positive implementation climate decreases role ambiguity by expecting, supporting, and rewarding their roles as counselors in particular rather than penalizing teachers for shifting time away from teaching responsibilities. Examples of experiences we heard in a qualitative study with the first sequence included teacher counselors being able to gather extra chalk and paper to use in the group and fellow teachers reminding children about the group (to improve attendance) and helping prepare the room for group delivery. Another example was leaders stopping by and supporting supervision for teacher-counselors. When the counseling role is valued and prioritized, prior work has suggested that this reinforces a sense of purpose or satisfaction in the work, in turn reducing emotional fatigue [90,91]. A 2021 study in community mental health found that role clarity is the leading factor influencing quality of care and lessening emotional exhaustion, making it a critical area for organizations to address in improving provider well-being and care [92].

The second pathway that accounted for low STS among teachers involved both a strong supervisory relationship and a positive organizational climate via their perceived work environment. These organizational factors together may have nurtured both relational and workplace support to buffer teacher-counselors from STS. A positive perceived work environment refers to counselors’ satisfaction with their organizational environment (e.g., workplace morale, cohesion, learning space), which may reinforce belongingness in the organization. For teachers navigating multiple responsibilities and roles, this combination of interpersonal support for the counseling role via supervision and a healthy work environment as a whole may reduce the emotional burden that leads to STS by making their lay counselor work feel manageable both clinically and emotionally and valued by the workplace [76,93].

The final pathway among teachers revealed that low transactional leadership in combination with high transformational leadership was sufficient for low STS. This may suggest that in structured environments like schools, where teachers already manage multiple responsibilities, additional transactional leadership, which focuses on performance and reward-based motivation, may not provide added benefit and could even amplify stress as shown in previous research [94]. In such settings, the absence of transactional management may allow transformational leadership to emerge more fully. Transformational leadership is characterized by leaders who inspire, motivate, and support the development of their staff and foster a sense of shared purpose and meaning. Several studies have pointed to an indirect relationship between STS and transformational leadership. Strolin-Goltzman et al. (2020) found a significant relationship between high transformational leadership and low STS among mental health providers working in the US child welfare system [34]. Another study in child welfare agencies, where providers are embedded into an organizational system like teacher-counselors in the present study, found that transformational leadership mediated the relationship between STS and turnover intention [95]. It is important to note that this pathway differs from the CHV context, where high transactional leadership was present in predicting low STS. In that context, transactional leadership may provide needed structure for CHV-counselors operating more independently, whereas teachers already work within a structured environment. These findings highlight the importance of context-sensitive leadership, where the same leadership style may have different effects depending on the provider’s organizational needs.

### Strengths and limitations

A key strength of the present study is the focus on organizational factors related to STS among lay counselors in two distinct sectors. Moreover, the use of CNA as an analytic method to identify combinations of organizational factors that make a difference in whether low STS occurs among lay counselors when present together was especially valuable. CNA is a configurational comparative method designed to capture the equifinality that is often present in implementation science, where data is collected within complex interventions and real-world settings [96]. Rather than identifying factors that are independently predictive of outcomes like a regression analysis, CNA detects how different factors work together, which is especially important when studying outcomes like STS that are likely influenced by multiple intersecting factors. This approach offers practical, context-specific findings that can inform future implementation efforts, which is why CNA is growing in popularity within implementation science. Additionally, this study is one of the first to apply CNA to examine a workforce well-being outcome, rather than implementation or clinical outcomes, among lay providers in a resource-limited setting. This broadens the potential applications of CNA within global mental health. The study also benefits from parallel analyses conducted among teacher-counselors and community health volunteer (CHV) counselors, allowing for a more nuanced understanding of how organizational factors operate differently across contexts and roles.

There are also multiple limitations to note. First, the organizational factors we could examine were limited by the constructs captured within the parent study, BASIC. It is likely that there are other important factors that also contribute to low STS on an organization-wide level that were not included in this study, as well as on an individual level, including teachers or CHVs own emotional well-being before beginning to deliver PT. Given our focus on organizational support, it is also important to note that we cannot disentangle situations in which, for example, teachers with less resilience may be nested within schools with less organizational support. Analytically, while CNA provides valuable insight into necessary and sufficient conditions, it is a relatively novel method and results should be interpreted as exploratory to inform future work. Another limitation is the modest internal consistency of the STS measure. The dichotomization of this variable in CNA may have introduced some misclassification near the cutoff, which may bias findings toward the null; thus, pathways should be interpreted conservatively. Moreover, the outcome factor, low STS, had a high prevalence (72.6% for teachers and 85% for CHVs), which can skew the data to appear to have high consistency and coverage as the outcome is present with many configurations. However, this limitation was managed via the use of prevalence-adjusted consistency and coverage calculations in the analyses by imposing a stronger penalty when prevalence is high in consistency vice versa for coverage. The result is a more reliable evaluation when prevalence is high.

## Conclusion

This study adds to our understanding of how organizational factors can support lay counselors delivering trauma-focused treatments, whose long-term well-being is critical to sustaining mental health care for youth in resource-limited settings. Using CNA, the study revealed various organization-level pathways to low STS for teacher-counselors and CHV-counselors. While pathways differed across counselor/organizational types, and no single factor was universally necessary and/or sufficient for low STS, several themes consistently emerged: strong supervisory relationships, implementation climate and leadership supporting PT, feasibility for delivering PT, work environments, and transactional or transformational leadership. A key takeaway is that organizational strategies to prevent or mitigate STS should be tailored to provider roles and the organizational contexts in which they are embedded. As task-shifting models and the use of lay counselors continues to expand in LMICs and beyond, prioritizing counselor well-being in organizational efforts during intervention development is critical for the sustainment and effectiveness of trauma-focused mental health care. The present study can inform the appropriate use of organizational supports and future work should explore which combination of factors are important for low STS in similar resource-limited contexts with lay counselors.

## Supporting information

S1 Checklist Checklist.
(DOCX)
