# Supplementary material for: Organization-level determinants for low secondary traumatic stress in lay counselors delivering trauma-focused cognitive behavioral therapy in Kenya
Source: PLOS Glob Public Health. 2026 May 26;6(5):e0006360. doi: 10.1371/journal.pgph.0006360 (PMC13210311; doi:10.1371/journal.pgph.0006360)
Supplement: S1 Checklist — Checklist. (DOCX) [file pgph.0006360.s001.docx]

Inclusivity in global research

PLOS’ policy on inclusivity in global research aims to improve transparency in the reporting of research performed outside of researchers’ own country or community and ensures that PLOS publications reporting global research adhere to high standards for research ethics and authorship. Authors of relevant research articles may be asked to complete the questionnaire below, which outlines ethical, cultural, and scientific considerations specific to inclusivity in global research. This questionnaire may be requested when researchers have travelled to a different country to conduct research, if research uses samples collected in another country, research with Indigenous populations or their lands, or if research is on cultural artefacts. Researchers travelling to another country solely to use laboratory equipment will not normally be required to complete the questionnaire. However, the questionnaire can be requested at the journal’s discretion for any submission – if you have been requested to complete this questionnaire by the PLOS journal you submitted to, please do so.

Please complete the questionnaire below and include this as a Supporting Information file with your manuscript. Note that if your paper is accepted for publication, this checklist will be published with your article in the supporting information files. Please ensure that you reference the checklist in the main body of your manuscript. We suggest adding a subsection ‘Inclusivity in global research’ to your Methods section and adding the following sentence: “Additional information regarding the ethical, cultural, and scientific considerations specific to inclusivity in global research is included in the Supporting Information (SX Checklist)”

The questions have been designed to be applicable to a wide range of study types, and there are subsections for both human subjects research and non-human subjects research. If any of the questions are not relevant to your research please mark them as “N/A” as appropriate.

**Ethical considerations, permits and authorship**

*This section is applicable to all research types.*

Provide details as to who granted permissions and/or consent for the study to take place in the Methods section of your manuscript. This should include the names of **all** ethics boards, governmental organizations, community leaders or other bodies that provided approval for the study. If individuals provided approval refer to these people by their role or title but do not list their name(s).

Reported on page number: Page 10

If there were any deviations from the study protocol after approval was obtained please provide details of these changes in the Methods section of your manuscript.
Did this study involve local collaborators that are residents of the country where the research was conducted or members of the community studied? If you do not have any authors from said communities, please provide an explanation for this below.

Reported on page number: Page 11

Yes, the study did involve local collaborators that are residents of the country where the research was conducted. Two trainers involved in Trauma Focused Cognitive Behavioral Therapy Train-the-Trainer training and mental health consultation were involved in the development and writing of this manuscript, and they are authors (Daisy Anyango Okoth, Omariba Anne Nyaboke). The Kenyan study PI and Ace Africa Director, Augustine Wasonga, would also be an author (he is on all other manuscripts until this death in January 2024).

Everyone listed as an author should meet PLOS’ criteria for authorship and all individuals who meet these criteria should be included in the author byline, rather than the acknowledgements. For further information please see the journal’s Authorship Policy.

**Human subjects research (e.g. health research, medical research, cross-cultural psychology)**

Did you obtain written informed consent from a representative of the local community or region before the research took place? How did you establish who speaks for the community? Details of written informed consent obtained from study participants should be reported separately in the Methods section of your manuscript.

The research questions that are the focus of the parent study, BASIC, came from the NGO we collaborated with, Ace Africa (led by Kenyan PI, Augustine Wasonga). After seeing how effective the culturally adapted TF-CBT was in their community (with Ace Africa employees as lay counselors) Ace Africa decided that for sustainment, teachers or CHVs would deliver TF-CBT, given Ace Africa’s existing collaborations with both schools and health facilities. We did not officially obtain written informed consent from a local community representative, but Ace Africa –led by and employing Kenyans- developed the questions and determined the partners. Ace Africa leaders (all of whom are Kenyan (with Mr. Wasonga a former teacher himself) decided who spoke for participating communities (usually head teachers and deputy teachers in schools; elders and chiefs in villages, and CHEWS in health facilities).

How did members of the local community provide input on the aims of the research investigation, its methodology, and its anticipated outcome(s)?

The BASIC study was developed based on Ace Africa’s experiences and requests from the Bungoma Region community and officials (as teachers and community members had seen the benefits for children and families). Prior to writing the BASIC study proposal for study funding and implementation, mPIs Dorsey and Whetten traveled to Bungoma to inquire as to whether there was interest in conducting an “implementation science study” that would look at organizational practices and policies that would support successful implementation of the culturally adapted TF-CBT. Due to the success of the previous RCT of Pamoja Tunaweza, government officials and community members were enthusiastic about an implementation science study. The mPIs  had as a goal of the pre-proposal writing visit of seeing if there was a desire to continue the work and, if so, if there was an implementing partner who wanted to be involved. To the surprise of the mPIs, there were 3 implementing partners who wanted to be involved in the new study – the Ministries of Education, Health, and the Red Cross. We decided to move forward with the Ministries because of the potential of long term sustainability of the intervention. In addition to these meetings, the implementing partner organization had a community advisory board that was specific for studies related to vulnerable children where were actively involved in suggesting implementation ideas.

When engaging with the local community, how did you ensure that the informed consent documents and other materials could be understood by local stakeholders?

Local stakeholders reviewed the original survey instruments. The local partner organization, Ace Africa, reviewed the informed consent documents. All documents were professionally translated into Swahili and then translated back into English by a professional who did not have the original English versions. These documents were then compared by the local study personnel and the Duke University-based research leads and the Duke University Institutional Review Board to ensure the accuracy of translations.

Will the findings of the research be made available in an understandable format to stakeholders in the community where the study was conducted (e.g. via a presentation, summary report, copies of publications, etc.)? Please provide details of how this will be achieved.

PI Kate Whetten is planning to return in 2026 to report back to National and Local Government officials and NGO networks on the results of all BASIC findings, including this manuscript, via presentations and reports. MPIs Whetten and Dorsey are also working with co-author Daisy Anyango Okoth to disseminate across social media platforms. A new NGO, TINADA, is predominantly a youth mental health organization and seems to have a good reputation in the NGO networks for East Africa. TINADA has a Kenya only organization as well as a separate Africa-wide organization with almost the same name. They created the separate org for multi-country work. They can also post a link to this manuscript, when published on theie website, with a 1-2 sentence description.

**Non-human subjects research using specimens/ animals collected as part of the study, or those housed in archival collections. Examples include archaeology, paleontology, botany and zoology.**

Did the permission you obtained from a local authority to perform the study include an agreement on access to outputs and benefit sharing? This may include procedures to enable fair distribution of the benefits and resources arising from the research performed. Please include any details of Prior Informed Consent and Benefit Sharing Agreements obtained. These may be required by field-specific regulations, for example the Convention on Biological Diversity (CBD) and the associated Nagoya Protocol.

If the material used in your study was imported, please A) provide the year it was imported and B) indicate whether permits were obtained to import/export the materials used, C) provide details of any permits obtained. If this information is not available, please indicate this.

If you used archival specimens, please state how the material used in your study was acquired by the institute it is held in and provide details of any permits obtained for the original excavations/ sample collection. If this information is not available, please indicate this.

How was the potential cultural significance of the materials collected in your study to local communities considered in your research design? Were Indigenous peoples and/or local researchers and institutions involved with archaeological excavations / collection of specimens? If so, please provide a description of their involvement.

If your manuscript includes photographs of human remains please indicate whether authors obtained permission from descendants or affiliated cultural communities to do so.
